# Progress in Surgery

**Published:** 1900-05-26

**Authors:** 


### PROGRESS IN SURGERY.

### DISEASES OF KIDNEYS.

**Cystinuria.**—Moreigne[1] believes that cystin is excreted only when the metabolism of the body is below normal and oxidation deficient. Uric acid is also turned out in large quantity, the urine being pale, and of a greenish yellow colour. The cystin present is deposited as crystals, and with it is generally found tyrosin. At a recent meeting of the Berlin Medical Society Cohn reported a case of cystin calculus in a girl of seven and a half years. The mother of the patient and six of her brothers and sisters were subjects of cystinuria.

**Bacterinuria**, according to Predohl,[2] occurs most often in women, and is marked by burning and scalding near the end of micturition and lumbar pain. The urine is cloudy, generally without any special odour, acid, and does not clear up when heated; sometimes, however, it is very fœtid. The organisms which form the cloudy deposit are usually the bacterium coli alone, and it is difficult to render the urine quite free from them. Salol, benzoate of soda [and urotropine] have been recommended for this purpose. Bruce Clarke[3] points out that some authorities think the bacillus coli alone invades the urine, but others hold that pus cocci do so frequently. Personally, he believes that cocci are often the first comers, but their presence paves the way for the bacilli who soon supplant them. T. L. Maxwell has carried out experiments the results of which support this view. Phosphaturia has been the subject of a paper by Klemperer,[4] who points out that the condition arises when much acid is removed from the system by secretion into the stomach, especially if vomiting takes place, or a vegetable diet is used.

In neurotic patients there is often gastric catarrh and dyspepsia which lead to a deficiency of acid and the consequent deposit of phosphates. Hence it may be as important to overcome these disorders as to prescribe a meat diet in place of a vegetable one. Another condition dependent on a reduction of the hydrochloric acid present is indicanuria. Allen A. Jones[5] shows that fermentation resulting in the formation of this substance may easily take place in ileus, stricture of the small intestine, carcinoma even without stagnation of the stomach contents, achylia gastrica, and inflammatory states of the intestines.

In a discussion on proteids in the urine Halliburton[6] pointed out that the presence of much globulin as opposed to serum albumin indicates a serious disorder, since it is only when the epithelial cells are much damaged that the large globulin molecules can pass through. The still larger molecules of fibrinogen are rarely able to get through at all. Among substances with small molecules pepton in the urine depends on bacterial digestion in the body and the excretion of the products through the kidneys. The substance is really dentero-proteose. Bradshaw reported a case of myelopathic albumosuria, the twelfth on record. The patient was a man of 70 years, with disease of the bones. The urine was milky, gave a precipitate on heating or with acid, which chiefly dissolved on boiling. The substance seems to be an albumose not found elsewhere, and its appearance is the first indication of the bone disease.

The so-called hæmorrhage from a healthy kidney in young adults has been studied by E. Hurry Fenwick.[7] He localised the bleeding in one kidney by the use of the cystoscope in two cases, opened the pelvis and examined it under a strong light. There he found a bright red engorged papilla covered with a meshwork of dilated vessels. This he suspects to be due to a small patch of interstitial nephritis impeding the reflux of blood, and claims that a cure of the bleeding can be effected by scraping the affected papilla.

**The Effect of Anæsthetics on the Kidneys** has been investigated by R. Coleman Kemp,[8] who states that ether produces a special contraction of the renal arterioles, with a damaging effect on the secretory cells as if the artery had been clamped. Albuminuria and even suppression may follow, and he thinks it ought not to be used when renal disease is present. The effects of chloroform on the kidney are practically nil, the A.C.E. mixture has the disadvantages of both anæsthetics. Anæsthol and Schleich's mixture are also dangerous. The least injury to the kidneys is done by nitrous oxide and oxygen. Galeazzi and Grilli[9] have tested the influence of anæsthetics on renal permeability. In healthy subjects they found that the colour of methylene blue appeared in the urine in half-an-hour, and continued increasing for three hours, disappearing in 50 or 60. A retardation was caused by the administration of ether, and still more by that of chloroform.

**Nephritis from Injury** is little known, and may be either parenchymatous in form, or suppurative and interstitial. T. J. Yarrow[10] describes a case of the former in a boy of seven, who had been run over, and in whom acute nephritis came on and lasted for one month. An interesting feature in the case was the presence of hæmatoidin in the urine until the blood clot was removed from the contused kidney. This, he thinks, is derived from hæmoglobin, which becomes hæmatin, and finally, by further oxidation in contact with the tissues, it is converted into hæmatoidin.

**Tuberculous Disease of the Kidneys.**—D. Newman[11] holds that there are three forms of this disease—(*a*) acute miliary, (*b*) local tuberculosis, and (*c*) caseous nephritis. Owing to the remarkable immunity of the kidney bacilli are often excreted through it without injury. Hence their presence in the urine is no evidence of kidney mischief. Various diseases and injuries may render the kidney liable to suffer from passing bacilli, which gain access to it—(*a*) by the blood stream, (*b*) by lymphatics, (*c*) up the channel of the ureters, and (*d*) [theoretically] by extension from neighbouring organs. Miliary tuberculosis is always associated with disease elsewhere, most commonly in the lungs, often in the meninges, and but rarely in the prostate or bladder. Indeed, it does not extend to the bladder for some time afterwards, and there are, therefore, few or no symptoms, such as pain or dysuria. Tuberculous embolism may result in small localised, or even encysted, areas of disease favourable for surgical interference. In some cases of renal tuberculosis it is clear that spontaneous cure may occur, at least to the extent of the disappearance of tuberculous material and the substitution of fibrous tissue for it.

Bolton Bangs gives the results of operative interference in 135 cases. There was a mortality of 20 per cent. up to a month after operation. Newman advises nephrectomy when the disease is extensive, or where incision and drainage is not hopeful, or where a sinus does not heal up after nephrotomy. Indeed, nephrectomy at as early a period as possible is the operation most likely to give a permanent cure. Among medical measures for vesical irritation he recommends salol, benzoate of ammonia, antipyrin, with hyoscyanus. For alkaline urine calomel given by the mouth is often helpful, while washing out the bladder is generally harmful.

An instance of death from suppression of urine due to the irritation of calculi in a patient who had only one kidney is given by Percival Wood.[12] The patient remained at work feeling quite well for three days after suppression came on; on the seventh day when seen there was slight muscular rigidity and dulness in the right flank. Operative interference by Mr. D'Arcy Power failed to relieve him, and he died next day without developing any uræmic state or losing consciousness. It was found that the left kidney with its artery and vein were absent; the right was granular and contained four calculi.

[1] Med. Rec., Nov. 4. [2] Med. Rec., Dec. 16. [3] Lancet. [4] Med. Rec., Dec. 2. [5] Brit. Physic., Mar. 15. [6] Lancet, Feb. 24. [7] Brit. Med. Jour., Feb. 3. [8] New York Med. Jour., p. 732, 1899. [9] Brit. Med. Jour., Nov. 11. [10] New York Med. Jour., Jan. 6. [11] Lancet, Feb. 24. [12] Lancet, Jan. 6.

